# Variola, or Small-Pox

**Published:** 1838-05-23

**Authors:** N. Chapman

**Affiliations:** Professor of the Theory and Practice of Physic, in the University of Pennsylvania


### THE MEDICAL EXAMINER.

DEVOTED TO MEDICINE, SURGERY, AND THE COLLATERAL SCIENCES.

EDITED BY J. B. BIDDLE, M. D. AND M. CLYMER, M. D.

No. 11.] PHILADELPHIA, WEDNESDAY, MAY 23, 1838. [Vol. I.

### VARIOLA, OR SMALL-POX.

*By* N. Chapman, M. D., *Professor of the Theory and Practice of Physic, in the University of Pennsylvania.*

[Reported for the Medical Examiner.]

My attention is next to be directed to the consideration of eruptive fevers, commencing with variola, or small-pox. This is a disease of modern times; no account of it is to be met with in any of the writings of Greece or Rome, which have descended to us. Endeavours have been made by Willan and others to trace it to antiquity. But, elaborate and recondite as were their researches, they have not produced any satisfactory evidence of its existence, and the fact, as stated, is now sufficiently conceded. It has, on this point, been forcibly urged by Sydenham, Mead, and Freind, that, since Hippocrates, and, especially, Celsus and Galen are silent in regard to it, the works of the two latter being a sort of a digest of the knowledge of their predecessors, we are entitled to this conclusion, and the more so, from the precision of their history of diseases, that no such had occurred, or with which they were acquainted.

The earliest notice of it is by the Arabian writers. An old manuscript in the library at Leyden, dated 572, declares, that, "in this year, small-pox and measles made their appearance in Arabia." It seems, however, that, several years before, it broke out at the memorable siege of Mecca, where it raged with great violence in the Christian army, leading to its total discomfiture. This event happened, according to Gibbon, the historian, two months prior to the birth of Mahomet.

To Rhazes, a most distinguished man, who lived at the commencement of the 10th century, we are indebted for the first full and accurate description of small pox; and he states, that it was brought out of Ethiopia into Arabia. To the writings of his predecessors long since lost, by whom it had been previously noticed, he, however, refers, and, especially, to those of Ahron, a physician of Alexandria, in Egypt. The latter resided in that city, in 641, when attacked by the Arabians under Omar, the successor of Mahomet, and it is not improbable, the disease was conveyed to it by the invading army, and, in this way, he became conversant with it. Comparing dates, we shall find, that this was seventy-nine years after the siege of Mecca, on which occasion, so far as ascertained, small-pox, as I have said, sprung into being.

Nevertheless, by some it is supposed, that it originated in China, or the remoter India, or that, at least, it was known in these regions for centuries anterior to the period I have mentioned. But medical writings not existing, or to which access can be had, among these people, this opinion, not resting on authentic records, is a mere deduction from their mythology, religious institutions, some allusions in their civil history, their traditions, and other sources equally vague and inconclusive.

Considering, however, the intimate connection of the Arabians with the East, they might have derived it from that quarter of the world. Be this as it may, we have the most satisfactory proof of its introduction and diffusion through Spain, Sicily, and the Levant, by the Saracens, when they, in the eighth century, overran these countries. But though it had thus gained a partial admission into Europe, it did not generally, till the close of the twelfth, or the beginning of the thirteenth century, when the Crusaders were engaged in the Holy Wars.

Contracting the contagion in Palestine, these bold and enthusiastic adventurers introduced it, on their return, into their native places. The intercourse of the nations of Europe with each other, becoming greatly extended by commerce, about this time, it spread rapidly throughout Christendom, and, for several hundred years, its ravages were terrible. Nor were these, subsequently, in our own hemisphere less extensive in proportion to the number of subjects. Conveyed to it by the successors of Columbus, the tale of the misery and desolation it inflicted is painful to peruse. Twenty-five years after the discovery of this continent it occurred, and we are told, that it destroyed more than a moiety of the population of the provinces into which it penetrated. Three millions and a half are computed to have fallen victims to it, in a very short time, in the kingdom of Mexico alone.

Brought afterwards by emigrants from Europe to our immediate land, it swept off also, several tribes of the aborigines, leaving scarcely a sufficiency of them to preserve their name. Gradually it reached other and obscurer regions, owing to the enterprises of discovery and exploration, or to the slower encroachments of civilization, till very few portions of the globe, perhaps, can now claim an exemption from it. There is, indeed, only a single exception in the universality of its pervadence of which I am aware. It is stated, on the best authority, "that no case of small-pox, measles, or whooping cough, has been met with in New South Wales,"* which is the more remarkable, from the freedom of intercourse with that colony.

Not having existed in the classic ages, there could be no term for it in the Greek or Latin languages. But as some appellative designation was required for the disease, Variola was coined from either the Latin word *Varius*, signifying *spotted* or *speckled*, or *Varus*, a *pimple*. The vernacular title is said to be derived from the Saxon *pocca*, a pock or little pouch, and the epithet *small*, *variola minuta*, was afterwards adopted to distinguish it from syphilis, when it appeared, then vulgarly called great pox.

* Evidence before the Committee of Emigration of the House of Commons.

The first case on record of *Variola*, by this name, is that of Elfrida, daughter of Alfred of England, and wife of Baldwin the Bold, Earl of Flanders, and the next, that of her grandson, Baldwin. The one occurred in 907, and the other in 961. These cases are interesting as showing, that the disease had crept into the west of Europe at an earlier date than is generally stated. Nor, probably, were they the only instances of it. Destitute of medical writers at the time, there was no regular history of the disease, and it is presumable, that these two cases were singled out by the monkish annalist who relates them, as notable events, from the superior rank of the personages in whom they happened.

It has been usual to divide the variolous disease into two species, or varieties, according to the appearance of the eruption, the distinct or discreet, and confluent, or, when the pustules are separate, or, with intervening spaces between them, and when they run into each other and coalesce, so as to form nearly an undistinguishable mass. But this is an arbitrary division, it often happening, that while the eruption is distinct in one, it may be confluent, in other portions of the body. It is also wrong to characterize a disease by a single incident, however prominent it may be, and, especially, when it is fluctuating, and liable to diverse modifications. Besides which, the distinction is founded entirely on an external appearance, that has comparatively little connection with the real pathology of the disease. As variola is attended by fever, or a general condition, either inflammatory or congestive it seems to me, it were better, to treat of it, at least as concerns practical advantages, in conformity with these views, and, hence, I shall adopt such an arrangement.

Nothing very peculiar is discernible in the introductory symptoms, of the first or inflammatory small-pox. Like pyrexia, generally, it commences with languor, weariness, aches in the head, back, and lower extremities, chilliness, alternated by flushes of heat, thirst, nausea, or vomiting, præcordial uneasiness, pain in the epigastrium, and some rigidity, or soreness of throat. Fever being developed, the pulse becomes full and active, the skin warm, the face turgid, the eyes slightly injected, the perspiration hurried, the tongue white, or sometimes red at the point or edges, the stomach still irritated, and betraying tenderness on pressure, the bowels costive, and the urine scanty, and high coloured. The irritation extending to the lungs or appendages, these betray the implication, by acute or dull pain, in some part of the chest, and by more or less embarrassment of breathing, according as the pulmonary substance or tissues, or the trachea, or its terminations may be concerned.

Continuing pretty much in this way, till towards the third day, some exacerbation of the febrile state is manifested, and now confusion of mind, or even delirium may occur, or there is heavy somnolency only. The epigastrium is exceedingly tender, the vomiting more violent, the tongue very florid, the hands and feet cold, while the surface, generally, is hotter, with a singularly copious perspiration, emitting a very peculiar and offensive odour. Nor is the pulmonary disturbance less heightened, by an increase of the catarrhal, laryngetic, pleuritic, or peripneumonic affections. During this period, hæmorrhage from the nose is apt to occur in adults, and convulsions in children, in whom this further peculiarity may be remarked, that they perspire less, and have not, in an equal degree, the smell to which I have alluded. An exasperation of symptoms is usually the immediate precursor of the eruption.

Breaking out, as minute red specks, about the end of the third, or early on the fourth day, first on the face, particularly on the forehead, nose, around the mouth, then on the neck, and wrists, the eruption extends over other parts of the body, the chest, abdomen, and back, lastly on the lower extremities, and is completed in forty-eight hours. From the commencement of the eruption, the fever gradually abates, and, with its completion, entirely subsides.

These red specks, at the close of the second day, become a little elevated, having a slight central depression, and an inflamed base. Towards the fourth day they undergo a further change. Now may be perceived in them, a small portion of limpid fluid, as in a vesicle; thence they enlarge and become more conspicuously depressed in the middle. An inflamed circular margin or areola, of a rose or damask colour surrounds each, which, the eruption being considerable, spreads, and runs into each other, occasioning more or less increase of tumefaction, especially of the face and eyelids. By the seventh or eight day, the vesicles having further augmented in size, they assume a more spheroidal shape, and begin to fill with purulent matter. It is conjectured, though not satisfactorily, that the vesicle is thus gradually converted into a pustule by the absorption of the pellucid fluid, and the secretion of pus in place of it, or by the former being changed into the latter. This suppurative process continues for three or four days, the pustules growing still larger, fuller, more yellow, and opaque, till they attain maturity, which is generally on the twelfth day, and now, from extension, lose their central depression, or at least, most of them undergo this change.

It is at this period, when the eruption is extensive, that the secondary fever, as it is called, arises, owing to the irritation of the skin, which subsides with the cause of it. There is, simultaneously with the fever, an aggravation of the soreness of throat, and difficulty of swallowing the saliva, and fluids of the fauces, which become very viscid, creating a constant seriatus, or hawking, or a copious salivation ensues, especially in grown persons—and the voice is hoarse, with other evidence of laryngeal irritation.

Little alteration immediately takes place in the eruption, and, sometimes, it remains stationary for several days. But, more commonly, a dark spot is soon discernible in the centre of the pustules, and, with this appearance, they begin to shrink, and dry away, till scabs are formed, which, falling off, leave a red surface, that gradually disappears, or pits, or scars, which permanently endure. In the decline of this state of things, the same order is observed, as in the rise and progress of the eruption—first decaying on the face, and so, successively, as it appeared.

Conformably to the preceding account, the career of this disease is distinguished, by four different stages, the invasive or febrile, the eruptive, the maturative, the declinative or scabbing, between each of which stages, there is an interval, averaging from three to five days. This is the usual character of inflammatory small-pox, where distinct, though it is often infinitely milder, and more benignant. Even this form of it, however, is subject to great varieties in many other respects, and which are made the basis of numerous species by the nosologists. It is impossible, within my limits, to notice in detail, these diversities, and modifications. No eruption whatever, in some cases, follows the fever; there are others, in which the form and contents of the pustules are widely different, and hence, the distinction of vesicular, vesiculo-pustular, crystalline, watery, siliquose, varicose, and horny pocks, &c. &c.

To these may be added other peculiarities; the first, where one or more pocks are included in another larger pock, or vesicle, the second, in which fresh pocks are formed on the tops of those previously existing, and a third, where there is only an efflorescence. These several varieties sometimes prevail pretty generally, though oftener individually, here and there a case presenting itself as a mere anomaly.

The disease being of an extreme typhoid or congestive nature, it sometimes begins with the manifestations of collapse, cold skin, pale and sunken countenance, great anxiety and oppression, and a very feeble circulation, which state may continue with little or no reaction. But more frequently, the invasion is indicated by languor and listlessness, dejection of spirits, heavy sighing, muscular soreness, and severe pain in the back and lower extremities, alternations of chilliness and flushes, great præcordial distress, and sickness of stomach. This state, which is usually protracted, is slowly succeeded by the development of fever, with a small, weak, quick pulse, unequal distribution of temperature, the head and trunk being extremely hot, amounting to even the "*calor mordax*," of the old writers, while the extremities are cool, the perspiration scanty and clammy, or the reverse, copious, and of a cadaverous odour. As the disease proceeds, it is marked by a gradual disclosure of cerebral and nervous disorder, giddiness, disposition to syncope, heaviness or absolute coma, subsultus tendinum, sometimes convulsions, free discharges of pellucid urine and watery diarrhœa, particularly if there be not vomiting.

The state of the epigastrium is not uniform, sometimes exquisitely tender on pressure, and is often otherwise, owing to the extinguishment of organic sensibility. Nor is that of the tongue, which is florid, and apparently raw, or heavily encrusted with dark sordes.

Generally, the eruption shows itself earlier, by a day or two, than the ordinary period, while, in other instances it is more delayed, even to the fifth, or sixth day, sometimes partially appears, and recedes, producing the most deadly sickness, syncope, or stupor, and convulsions. Taking place, however, the face is covered with small papular specks, which run into each other, forming a red, tumified rugous surface.

But, on some occasions, the primary appearance is that of an erysipelatous rash, or efflorescence. Little or no remission of fever is discernible on the occurrence of the eruption, and very often it is exasperated.

Great irregularity prevails in the further progress of the case. The eruption tardily advances, or reversely, very rapidly, so that the entire superficies is almost simultaneously covered. It may happen that the natural powers stop at this point, and no further effort is made towards pustulation, and the eruption becomes livid, or the whole recedes. But where it is otherwise, the pimples gradually enlarge, and fill with a thin, gleety, or darkish fluid, and rarely with yellow, consistent, purulent matter. These imperfect vesicles, instead of assuming a definite figure, with a flattened surface, and central indentation, are of every shape, sometimes conoid, or sunken, with ragged edges, coalescent, or interfluent, or if there be intervening spaces they are very pallid.

Every portion of surface is, at this time, swollen, the face, the eye-lids particularly, so much so, as to be closed, and the hands and feet, in scarcely a less degree. The eruption having reached maturity, the pustules are so blended, as to form one mass, or, in some parts, scarcely any separation can be discerned between them, constituting the confluent disease, which, though it may occur in the inflammatory, is much more incident to the congestive form of it. Extreme exhaustion not existing, an increase with the secondary fever of all the affections takes place.

To an aggravation of sore throat, and difficulty of swallowing, are added obstruction of the larynx, and much pulmonary and cerebral disorder, from heavy venous congestions of, or effusions in these organs. Cases of extraordinary malignity are, moreover, marked by petechiæ, vibices, colliquative hæmorrhages, especially bloody urine, with copious diarrhœa, and the pustules finally bursting, the matter escaping hardens into brown crusts, which fall off in ten or fifteen days, should life be preserved.

Of the malignant, there are as many varieties as of the more benignant disease, among which, may be enumerated, the erysipelatous, the morbillous, the miliary, sanguineous, and gangrenous or putrid, so called from the exhibition of such appearances, instead of those of the ordinary eruption.

(*To be continued.*)

